# Conversion surgery following severe cytokine release syndrome induced by immune checkpoint inhibitors doublet in advanced hepatocellular carcinoma

**DOI:** 10.1007/s12328-025-02155-6

**Published:** 2025-06-11

**Authors:** Haruki Mori, Hiromitsu Maehira, Nobuhito Nitta, Hajime Ishikawa, Toru Miyake, Sachiko Kaida, Katsushi Takebayashi, Masatsugu Kojima, Yusuke Nishina, Masaji Tani

**Affiliations:** Department of Surgery, Shiga University of Medical Science, Setatsukinowa-Chou, Otsu, Shiga 520-2192 Japan

**Keywords:** Cytokine release syndrome, Immune checkpoint inhibitors, Hepatocellular carcinoma, Conversion surgery

## Abstract

**Background:**

Combination immunotherapy, particularly the STRIDE regimen (durvalumab plus tremelimumab), is recommended as first-line treatment for advanced hepatocellular carcinoma (HCC). Cytokine release syndrome (CRS), while rare, can be a life-threatening adverse event associated with immune checkpoint inhibitors (ICIs). The relationship between CRS and antitumor activity remains unclear; however, some studies suggest that the occurrence of immune-related adverse events (irAEs) may be indicative of enhanced immune activation. We report a case in which severe CRS following ICI therapy led to marked tumor shrinkage and enabled conversion surgery.

**Case presentation:**

An 85-year-old woman presented with a 100 mm HCC in the right hepatic lobe with intrahepatic metastases, initially deemed unresectable. She received the STRIDE regimen and developed Grade 3 CRS with fever, hypotension, and multi-organ dysfunction. Corticosteroid therapy led to rapid clinical improvement. Three months post-treatment, imaging revealed tumor regression (from 100 mm to 60 mm) and significant decline in tumor markers (AFP 1550–110 ng/mL; PIVKA-2 32,600 to 79 AU/mL). Extended anterior sectionectomy was performed, with histopathology showing 90% tumor necrosis. The postoperative course was uneventful, and the patient was discharged on postoperative day 16.

**Conclusion:**

CRS is a potentially severe irAE that may also signal favorable immune activation. Early recognition and appropriate management of CRS are essential, and in select cases, ICI-induced CRS may facilitate curative conversion surgery in advanced HCC.

## Introduction

Hepatocellular carcinoma (HCC) is the third leading cause of cancer-related mortality globally and presents a significant public health challenge [1, 2]. For patients with unresectable HCC, systemic therapies are the standard of care. Immune checkpoint inhibitors (ICIs) have demonstrated efficacy across several malignancies, including HCC. The STRIDE regimen showed that combining durvalumab (anti-PD-L1) and tremelimumab (anti-CTLA-4) has shown promise in improving overall survival [3]. However, ICIs can cause severe immune-related adverse events (irAEs), which may pose significant challenges to treatment [4]. The advent of ICIs has also introduced the possibility of conversion surgery in select cases of unresectable HCC. This development represents a paradigm shift in treating advanced HCC, highlighting the growing importance of a multidisciplinary approach to achieve optimal outcomes [2, 5].

Cytokine release syndrome (CRS) is a rare immune-related adverse event (irAE), however CRS is a serious irAE that can lead to fatal outcomes. [6]. CRS activates T cells and macrophages, leading to an sudden increase in inflammatory cytokines such as interleukin-6 (IL-6), interferon-γ (IFN-γ), and tumor necrosis factor-α (TNF-α) [7]. The excessive release of cytokines can cause a wide spectrum of symptoms, ranging from fever, fatigue, and rash to hypotension and multi-organ failure, which clinically resemble sepsis [8]. Since the management of CRS differs substantially from that of sepsis, this similarity may lead to diagnostic delays and inappropriate initial treatment, potentially necessitating temporary cessation or modification of immunotherapy. CRS is particularly associated with immune therapies, such as ICIs and chimeric antigen receptor T-cell (CAR-T) therapies, which may complicate the clinical course and management [9].

We report an unresectable HCC treated with ICIs doublet, in which CRS developed. The condition was effectively managed with steroid treatment, leading to the resolution of CRS. ICIs doublet was effective for HCC, and tumor shrinkage was subsequently achieved, which enabled the successful performance of conversion surgery. 

## Case presentation

**Fig. 1:**
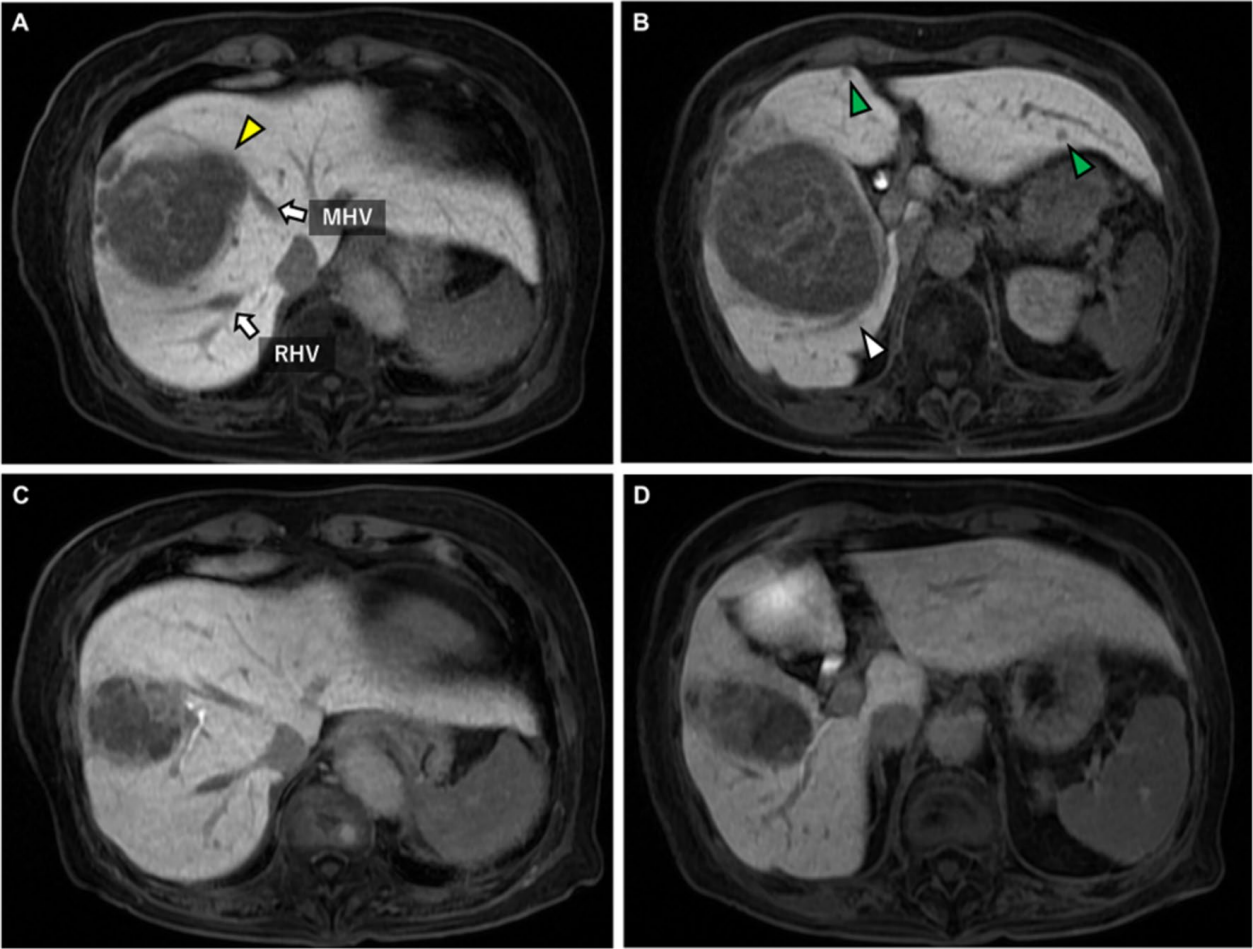
Magnetic resonance imaging (MRI). Huge hepatocellular carcinoma located in the hepatic anterior segment, before (**A, B**) and after (**C, D**) durvalumab plus tremelimumab treatment. **A**: The tumor caused compression and narrowing of the MHV (yellow arrowhead). **B**: Intrahepatic metastases in segments 3 and 4 of the liver (green arrowhead). Posterior segmental bile duct was dilated (white arrowhead). **C**: The tumor reduced in size. **D**: No intrahepatic metastases were found. *MHV* middle hepatic vein, *RHV* right hepatic vein

An 85-year-old woman with a medical history of hypertension was admitted to Shiga University of Medical Science Hospital (SUMSH) for further evaluation after being found to have liver dysfunction and a hepatic mass on abdominal ultrasound at a local clinic, in the absence of any specific symptoms. She had no drinking history and no hepatitis B or C. He had no family history of jaundice or hepatitis. A Magnetic resonance imaging (MRI) showed a 100 mm tumor in the right hepatic lobe and two smaller intrahepatic metastases in segments 3 and 4 (Fig. 1A). The imaging also showed narrowing of the middle hepatic vein (MHV) due to tumor compression and dilation of the posterior segmental bile duct (Fig. 1B). Laboratory studies showed elevated 1550 ng/mL of alpha-fetoprotein (AFP) and 32,600 AU/mL of des-gamma carboxyprothrombin (DCP). The indocyanine green (ICG) retention rate at 15 min was 16.3%, with a Child–Pugh score of 6 (grade A). The patient was diagnosed with Barcelona Clinic Liver Cancer stage B HCC. A right hepatic lobectomy combined with ablation therapy for two lesions in the left hepatic lobe was considered necessary for radical resection. However, the estimated residual liver volume was 398 mL, and the results of the ICG clearance test indicated that maintaining adequate postoperative liver function would be difficult. As a result, surgical resection was determined to be unfeasible. The patient was informed of the treatment options, including the combination therapy of atezolizumab and bevacizumab and the STRIDE regimen (durvalumab plus tremelimumab). After a thorough discussion and informed consent process, the patient chose to proceed with the STRIDE regimen.

The patient received tremelimumab (300 mg) combined with durvalumab (1500 mg) according to the NCCN Guidelines and the Japanese Society of Hepatology Guidelines. On day 2 post-administration, the patient developed fever (39.2 °C), right upper quadrant pain, and profound fatigue. Laboratory findings revealed leukocytosis (8700 μL) and elevated CRP (11.6 mg/dL). Despite empiric antibiotic treatment, her condition worsened, with hypotension and hypoxemia, leading to ICU admission on day 7 post-administration. Initially, infection-related symptoms were suspected, but blood cultures were negative. CRS due to ICI therapy was diagnosed at the time of ICU admission and was classified as Grade 3 according to the Common Terminology Criteria for Adverse Events (CTCAE). Corticosteroids (2 mg/kg) were administered, resulting in rapid symptomatic and hemodynamic improvement. 

**Fig. 2:**
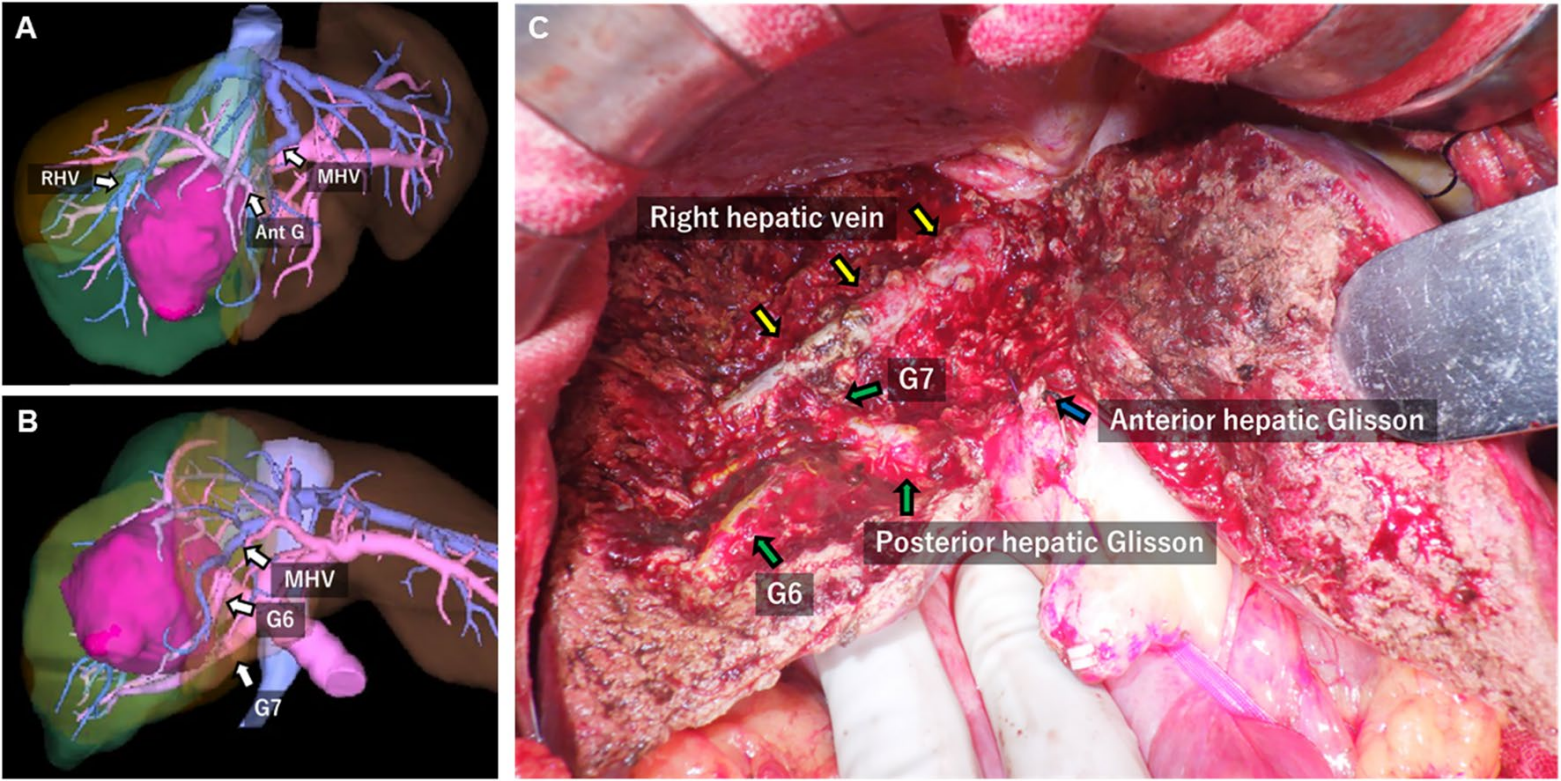
**A, B**: Preoperative asialoglycoprotein scintigraphy. Anterior segmentectomy was deemed feasible, with a resection rate of 27.0%. **C**: Intraoperative views of extended anterior sectionectomy with middle hepatic vein resection

Three months following the initial treatment, which consisted of a single dose of therapy with no subsequent courses, imaging revealed significant tumor shrinkage from 100 mm to 60 mm. In addition no evidence of dilation was observed in the posterior segmental bile ducts, and two intrahepatic metastatic lesions in the left hepatic lobe had completely disappeared (Fig. 1C, 1D). Tumor markers showed a significant decrease, with AFP reduced to 153.5 ng/mL and DCP to 222 AU/mL. Liver function tests, ICG clearance (15.3%), Child–Pugh score of 6 (Grade A) indicated preserved hepatic function. The liver resection rate was calculated by SYNAPSE VINCENT. For radical resection, an extended anterior sectionectomy with MHV resection was necessary. The estimated liver remnant volume was sufficient, measuring 649 mL, which accounted for 75.4% of the total liver volume (Fig. 2A, B). Conversion surgery was performed 4 months after the initial treatment, following the tapering of the corticosteroid dose to 7.5 mg/day. Intraoperative findings confirmed that the MHV was compressed against the tumor, consistent with the preoperative imaging. Therefore, an extended anterior resection with MHV resection was performed (Fig. 2C). The duration of surgery was 302 min, with a blood loss of 358 mL, and no intraoperative blood transfusions were required.

**Fig. 3:**
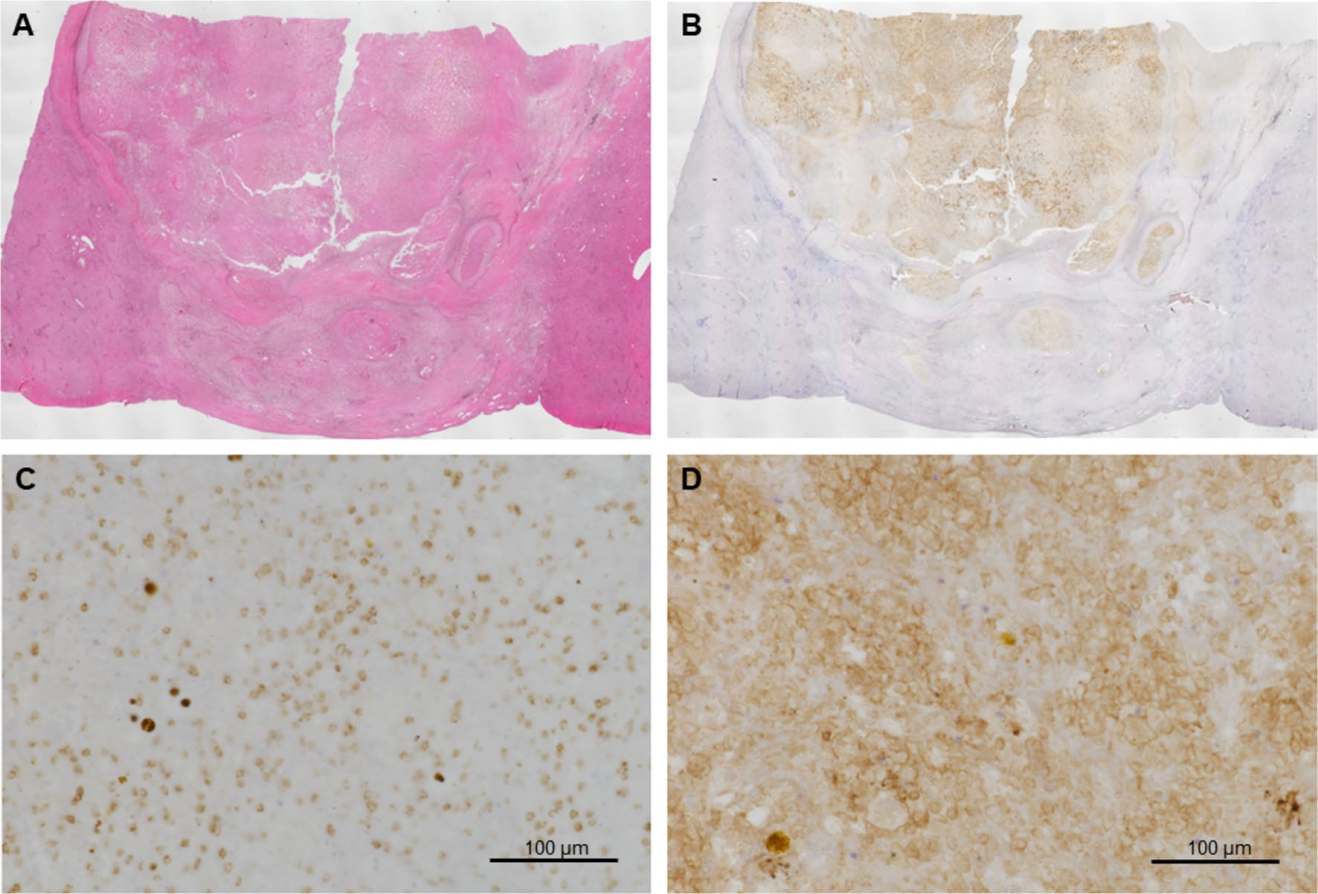
Histologic examination and immunohistochemistry. **A**: Hematoxylin and Eosin staining. PD-L1 (**B, D**) and CD8 + (**C**) expression by immunohistochemistry. CD8 + cytotoxic T-lymphocytes, PD-L1 programmed cell death ligand 1. Scale bar = 100 μm

The resected tumor was 60 × 50 mm in size, and the pathologic diagnosis was moderately differentiated hepatocellular carcinoma (HCC) (pT2N0M0 pStage II, vp0, vv0, b0, p0 UICC 8th). Histopathological examination revealed that 90% of the lesions were necrotic, with lymphocyte and plasma cell infiltration, indicative of changes associated with ICI treatment (Fig. 3A). No invasion into intrahepatic vessels or bile ducts was observed, and the resection margins were negative. Immunohistochemical staining demonstrated high expression of cytotoxic T-lymphocytes (CD8) and programmed cell death ligand 1 (PD-L1) within the lesions, whereas their expression in the liver parenchyma was low (Fig. 3B, 3C, 3D).

**Fig.4:**
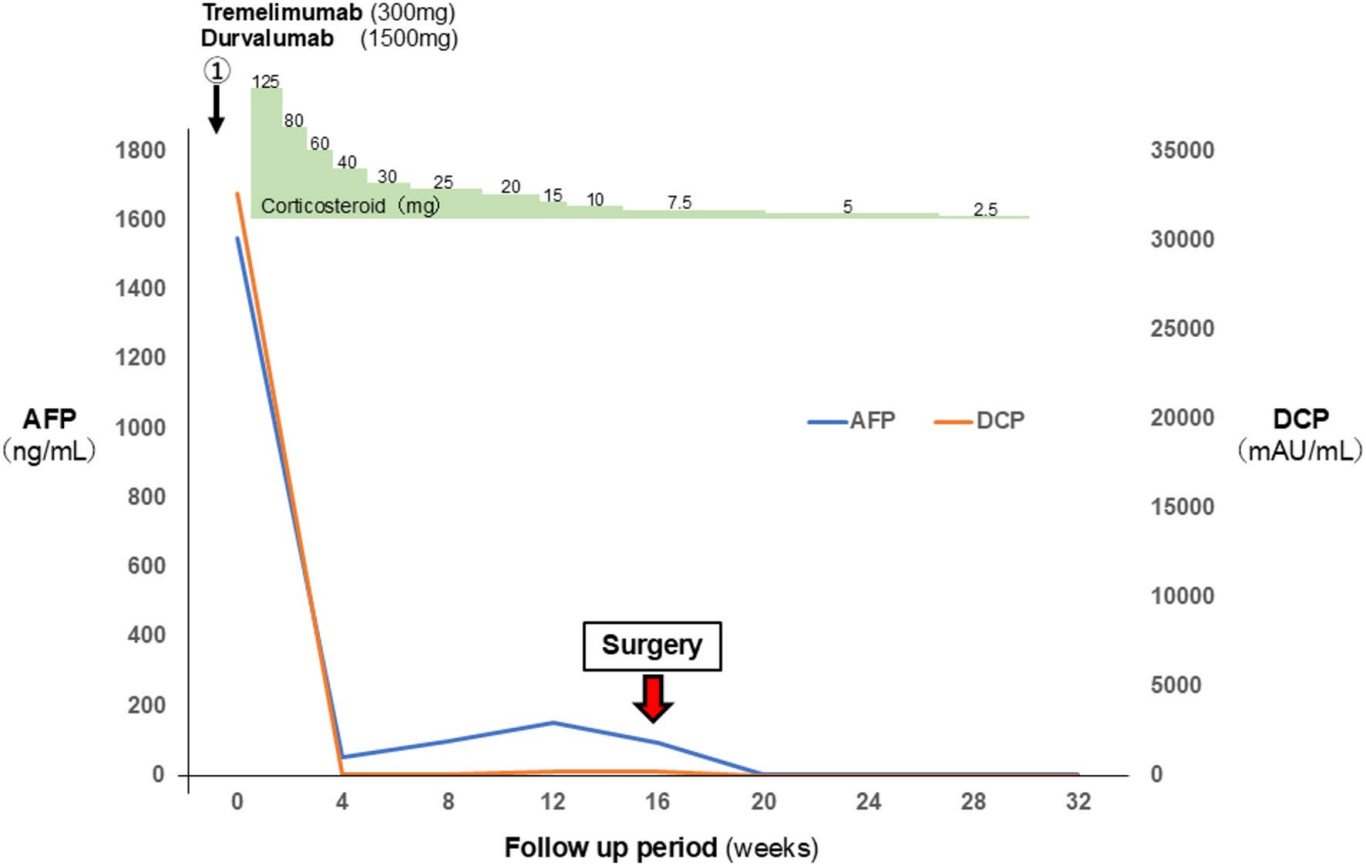
Perioperative course. The blue line indicates the change in AFP level, while the orange line indicates change in DCP level. Steroid therapy (2 mg/kg) was initiated following the onset of CRS and gradually tapered. Conversion surgery was performed 16 weeks after the initial administration of tremelimumab and durvalumab

The postoperative course was uneventful, with no complications. Rehabilitation was required for the recovery of activities of daily living, and the patient was discharged on postoperative day 16. Corticosteroid were gradually tapered and discontinued 30 weeks after initial administration. At present, 6 months after surgery, tumor markers remain within the normal range, and imaging studies reveal no signs of recurrence (Fig. 4).

## Discussion

This is the first reported case of conversion surgery following CRS induced by ICI doublet therapy in HCC. CRS is a well-recognized adverse event associated with T-cell engaging therapies such as CAR-T-cell therapy, however it is less frequently reported in the context of ICI therapies. ICIs activate immune responses against tumors but may also cause excessive immune activation, resulting in CRS [4]. HCC patients, who often have underlying conditions such as cirrhosis and chronic inflammation, may be predisposed to immune dysfunction and elevated production of pro-inflammatory cytokines within the tumor microenvironment [10]. Recent studies have explored the impact of irAEs and their management on overall survival in patients undergoing ICI therapy. Van Buren et al. [11] conducted a large cohort study demonstrating that irAE occurrence was associated with improved survival, and systemic corticosteroid use for irAE management did not diminish the survival benefits. However, early corticosteroid administration (within 2 months of ICI initiation) was associated with shortened survival, even when ICIs were continued. This suggests that while steroids effectively mitigate irAEs, their timing may influence long-term oncologic outcomes. These findings align with the case reported here, where corticosteroid therapy for CRS led to rapid clinical improvement and did not appear to compromise tumor regression or subsequent conversion surgery.

While the precise mechanism of CRS remains unclear, the hyperactivation of T cells and macrophages have been suggested to induce to the excessive secretion of inflammatory cytokines, including IL-6 and TNF-α by ICIs [12]. Recent studies have also implicated damage-associated molecular patterns (DAMPs), such as HMGB1 and S100 proteins, in the pathogenesis of CRS [13]. CRS symptoms often mimic sepsis, because both condition cause the similar symptoms including fever, fatigue, and hypotension, therefore it may take several time to differentiate between the two diseases. In this case, CRS was suspected only after sepsis was denied, emphasizing the importance of increased clinical monitoring during ICI therapy. These observations are consistent with a recent case reported by Ozaki et al. [14], in which CRS following durvalumab and tremelimumab therapy for advanced HCC was accompanied by elevated levels of IL-6 and DAMPs, including HMGB1. Their case also highlighted the diagnostic challenge of distinguishing CRS from sepsis and emphasized the importance of cytokine profiling.

Corticosteroid therapy is the standard treatment for CRS, in addition, IL-6 receptor antagonists, such as tocilizumab are also effective for severe or steroid-refractory CRS. Tocilizumab inhibits the IL-6 receptor, thereby modulating the immune response and alleviating CRS symptoms. Although tocilizumab is an established treatment for CRS, its potential impact on tumor progression in HCC remains unclear and warrants further investigation [15].

In this case, tumor regression enabled conversion surgery. However, there is limited clinical evidence on resuming ICI therapy following severe irAEs, necessitating cautious deliberation [16]. CRS is an uncommon but potentially fatal irAE. Identifying biomarkers to predict CRS risk and developing early prediction models based on the dynamics of inflammatory cytokines, such as IL-6, TNF-a and IFN-γ, are essential [17]. In addition, treatment algorithms tailored to CRS severity are needed to facilitate early diagnosis and prompt intervention [6]. These findings also highlight the need for further prospective studies to evaluate long-term oncologic outcomes in patients undergoing conversion surgery following ICI-induced irAEs. The integration of ICI therapy into surgical decision-making will require a multidisciplinary approach to optimize survival benefits while minimizing complications. Although re-administration of ICIs following CRS has been reported in selected cases, particularly when CRS is mild and well-controlled, the optimal strategy remains controversial. In this case, given the advanced age of the patient, severity of CRS, and sufficient tumor response to a single dose, additional ICI therapy was not resumed. Further studies are warranted to determine the safety and efficacy of ICI rechallenge after CRS in HCC patients.

This patient demonstrated sustained antitumor response despite ICI discontinuation, suggesting sufficient immunologic effects were achieved with the initial dose. The STRIDE regimen, involving dual blockade of PD-L1 and CTLA-4, significantly enhanced antitumor immunity, as evidenced by extensive tumor necrosis and lymphoplasmacytic infiltration. These findings indicate that even during CRS, immune-mediated tumor destruction was intensified. This case highlights the potential of ICI therapy to convert unresectable tumors into resectable candidates. Reports suggest that irAEs may correlate with improved clinical outcomes in ICI-treated patients, emphasizing the importance of effective irAE management [18]. The optimal timing of conversion surgery following immune checkpoint inhibitor (ICI) therapy in HCC remains a subject of ongoing investigation, as standardized criteria for treatment response assessment and the prognostic implications of conversion surgery have yet to be clearly defined. In this case, imaging confirmed tumor regression and liver reserve was reassessed before surgery. Tumor markers, such as AFP and DCP were instrumental in evaluating systemic therapy efficacy and guiding surgical decision-making. Immunohistochemical staining of the resected specimen revealed high expression of CD8 and PD-L1, even in necrotic tumor areas. However, preclinical models of NASH-induced HCC demonstrated that increased hepatic CD8 and PD-1 induced by immunotherapy impair immune surveillance without tumor regression, highlighting the need for predictive biomarkers for ICI efficacy [19]. Long-term oncologic outcomes following conversion surgery after ICI therapy remain unclear. Prospective studies are warranted to evaluate recurrence patterns, disease-free survival, and overall survival, ultimately refining patient selection criteria and optimizing treatment strategies for advanced HCC.

## Conclusion

This case highlights the risk of CRS in HCC patients receiving ICI therapy and underscores the importance of early diagnosis and management. As ICI therapy has become a cornerstone of systemic treatment for HCC, clinicians must remain vigilant for CRS and conduct comprehensive risk assessments tailored to patient backgrounds. Advancing our understanding of CRS pathophysiology and developing preventive and therapeutic strategies will be crucial for improving outcomes in HCC immunotherapy.
